# An abnormal left ventricular-atrial perforation after radiofrequency catheter ablation: a case report

**DOI:** 10.1186/s13019-019-1011-y

**Published:** 2019-11-04

**Authors:** Tingting Tao, Junnan Zheng, Hongfei Xu, Yiming Ni

**Affiliations:** Department of Cardiothoracic Surgery, The First Affiliated Hospital, Zhejiang University School of Medicine, 79#, Qingchun Road, Zhejiang, 310000 Hangzhou China

**Keywords:** Cardiac perforation, radiofrequency catheter ablation, complication

## Abstract

**Background:**

Cardiac radiofrequency ablation is a popular treatment for arrhythmias. However, it does have some complications, some of which are severe, even fatally. And there were limited reports on cardiac internal perforation after radiofrequency catheter ablation (RFCA) that required a surgical repair.

**Case presentation:**

A 47-year-old male was admitted to our hospital due to chest congestion for 4 months. He received a radiofrequency catheter ablation (RFCA) 9 months prior to admission. On admission, an echocardiogram showed an abnormal perforation between the left ventricle and the left atrium with moderate mitral valve regurgitation. We therefore performed a mitral valve replacement (MVR) and fixed the abnormal atrial-ventricular breakage via median sternotomy.

**Conclusions:**

Cardiac perforation is a severe complication of cardiac RFCA, operators should be extremely cautious to minimize radiofrequency associated perforations. Such a challenging and complex procedure should be deliberately considered by doctors and patients before implementation.

## Background

Cardiac radiofrequency ablation is a popular treatment for arrhythmias, and is said to have a very high success rate [1, 2]. Although, the method is proved to be feasible and effective in treating atrial fibrillation (AF) and other arrhythmia [3]. However, it does have some complications [1], some of which are severe, even fatally. But there were limited reports on cardiac internal perforation after radiofrequency catheter ablation (RFCA) that required a surgical repair [3]. Here we report a male patient with an abnormal left atrium-ventricle perforation after RFCA as well as moderate mitral valve regurgitation. We performed an open heart surgery and fixed the perforation.

## Case presentation

**Fig. 1:**
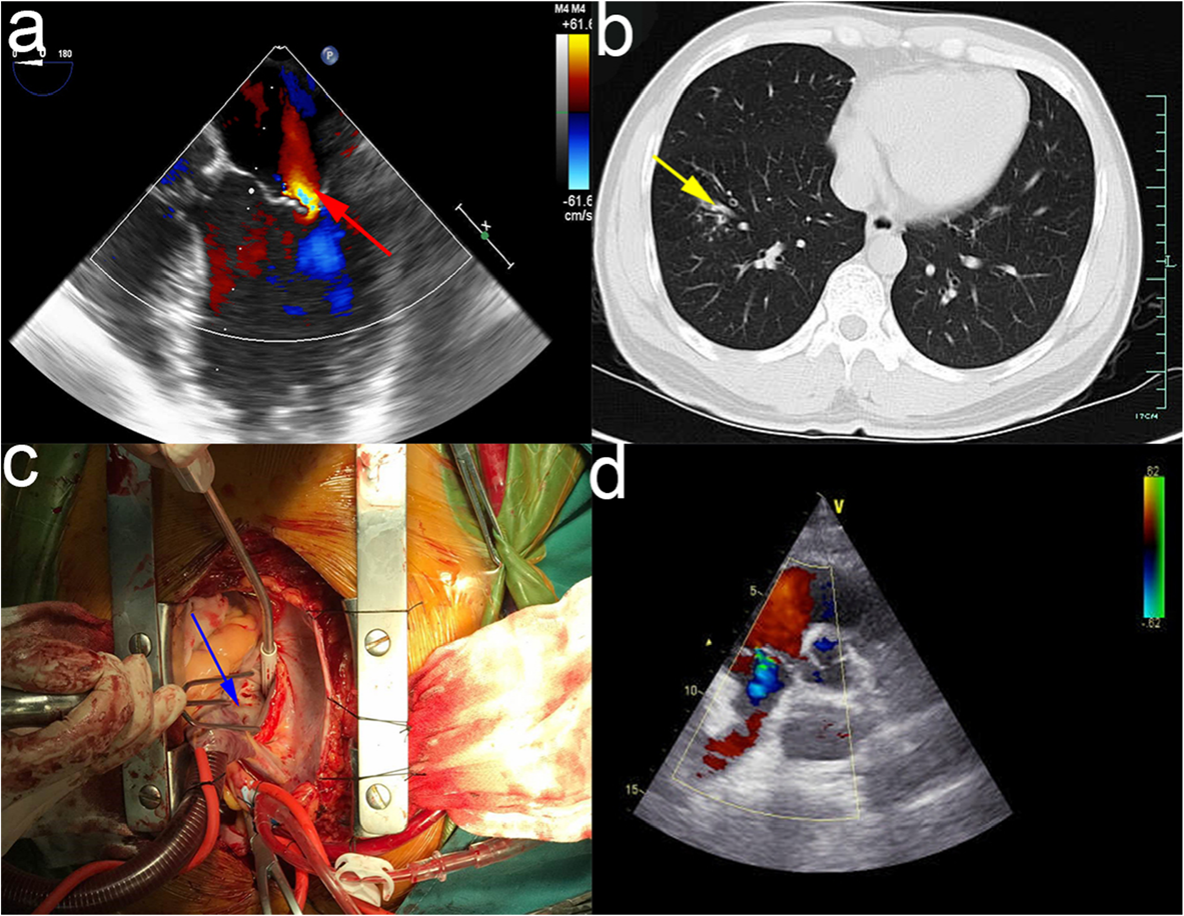
**a** The echocardiogram showed an abnormal perforation between the left ventricle and atrium with moderate mitral valve regurgitation. (the red arrow). **b** The CT scan showed there was bronchopneumonia in right lung lobe. (the yellow arrow). **c** The blue arrow indicated was exactly the perforation in the P2 area of mitral valve annulus, with a diameter of about 6 mm. **d** The post-operative echocardiography showed the mechanical valve worked well without any perivalvular leakage

A 47-year-old male was admitted to our hospital due to chest congestion for 4 months. Nine months prior to admission, the patient received a radiofrequency catheter ablation (RFCA) in local hospital due to paroxysmal supraventricular tachycardia (PSVT). The surgery was reported successful. However, the patient began to experience recurrent chest discomfort 5 months after the surgery. On admission, an echocardiogram showed an abnormal perforation between the left ventricle and the left atrium with moderate mitral valve regurgitation (Fig. 1a). And the CT scan depicted nothing abnormal of the heart but only bronchopneumonia in the right lung (Fig. 1b).

After completing relevant examinations and excluding obvious contraindication of operation, we performed a mitral valve replacement (MVR) and fixed the abnormal ventricular-atrial perforation via median sternotomy. During the operation, we found the perforation in the P2 area of mitral valve annulus, with a diameter of about 6 mm (Fig. 1c). Meanwhile, we found that the anterior mitral leaflet prolapsed seriously, and contracture were seen in both leaflets. At the first time, we tried repair the prolapsed valve using a 30# Sorin annuloplasty ring, however the leaflets coaptation was still not restored when being checked by injection test. Considering long term effect of the postoperative, we finally chose to have the mitral valve replaced with a mechanical prosthesis. We cut off the leaflets, placed a 27#St.Jude. mechanical valve and the closed the heart incision using 3–0 prolene. After all the surgical steps, the vital signs were stable, and the patient was sent to cardiac intensive care unit (CICU).

Postoperatively, echocardiography showed the mechanical valve worked well without any perivalvular leakage (Fig. 1d). The recovery of the patient was uneventful and he was discharged in a week.

## Discussion and Conclusions

Reviewing the echocardiography of the patient before surgery, the abnormal perforation in left ventricular-atrial was most likely caused by RFCA for the echocardiography before the procedure showed nothing wrong. And in our surgery, we performed a MVR and fixed the abnormal ventricular-atrial perforation via median sternotomy. At first, we did try a mitral valvuloplasty, however the coaptation height of the valve were only about 1~2 mm which was far from the required height of 4~8 mm, and the coaptation of the leaflets was still not restored when being checked with injection test. Considering postoperative long term effect of the patient, we thus chose to have the mitral valve replaced with a mechanical prosthesis. And the patient recovered well during follow-up.

Nowadays, cardiac radiofrequency ablation is a popular treatment for many kinds of arrhythmias [1], And the method is proved to be feasible and effective especially for symptomatic patients unresponsive to medical treatment [4], even announced as first line therapy for symptomatic supraventricular tachycardia [5]. However it does have some complications, some of which were even potentially life-threatening, for instance, cardiac tamponade. What’s more, some newly-presented complications associated with RFCA were identified, such as pulmonary vein stenosis and atrial-esophageal fistula [6]. Although this sort of major complications are rare and their rate is falling, an article published in 2012 reported that the overall complication rate after RFCA still ranged from 3.9 to 22% and major complications occurred in 3.9% of the patients [4]. Meanwhile the mortality of all patients who underwent RFCA procedures is about 0.1–0.2% [4]. We should improve our awareness to these complications and their causes. The reason may vary, it could be related with the use of high power or high temperature settings during radiofrequency delivery [2], or it might also be related to the site and type of ablation [1]. It is also reported that different anatomical and physiological circumstances might have the influence [2]. As RFCA is such a challenging and complex procedure, these factors should be carefully considered by both patients and physicians before surgery.

In addition, patients who are PSVT are possible to get tachycardia-induced AF after RFCA. And there is a study announced that whether remaining AF in post-ablation PSVT patients could be effectively predicted by the intra- and inter-atrial conduction delay [7]. It’s an overlooked etiology of PCIS since the widespread use of catheter-based procedures. Patients with small cardiac perforation during catheter ablation might develop PCIS, especially the elder or hypertensive patients.

Although cardiac perforation following a RFCA is rare, there is still 1% rate of ventricular ablation procedures. A timely surgical repair with cardiopulmonary bypass supported may help with the hemodynamic status of the patient. In this case, we emphasizes that these kind of uncommon complications associated with RFCA procedure should be paid attention to by the electrophysiologists.

For treatment, when a cardiac perforation is confirmed, a surgical repair is required for the majority of patients [8], and if a steam pop appeared and developed into cardiac tamponade, the surgical approach is even more in need [9]. It is also said that RFCA-associated pericardial effusion in patients treating arrhythmia is not rare and can easily lead to cardiac tamponade [10].

## Conclusions

Cardiac perforation is a severe complication of cardiac RFCA, operators should be extremely cautious to minimize radiofrequency associated perforations. Such a challenging and complex procedure should be deliberately considered by doctors and patients before implementation.

## Data Availability

The datasets used and analysed during the current study are available from the corresponding author on reasonable request.

## Abbreviations

AF: Atrial fibrillation
CICU: Cardiac intensive care unit
MR: Mitral valve regurgitation
MVR: Mitral valve replacement
PSVT: Paroxysmal supraventricular tachycardia
RFCA: Radiofrequency catheter ablation
